# Management of acute pancreatitis: current knowledge and future perspectives

**DOI:** 10.1186/1749-7922-1-16

**Published:** 2006-05-23

**Authors:** Lorenzo Fantini, Paola Tomassetti, Raffaele Pezzilli

**Affiliations:** 1 Department of Internal Medicine, Sant'Orsola-Malpighi Hospital, Bologna, Italy

## Abstract

In recent years, a number of articles have been published on the treatment of acute pancreatitis in experimental models and most of them concerned animals with mild disease. However, it is difficult to translate these results into clinical practice. For example, infliximab, a monoclonal TNF antibody, was experimentally tested in rats and it was found to significantly reduce the pathologic score and serum amylase activity and also to alleviate alveolar edema and acute respiratory distress syndrome; however, no studies are available in clinical human acute pancreatitis. Another substance, such as interleukin 10, was efficacious in decreasing the severity and mortality of lethal pancreatitis in rats, but seems to have no effect on human severe acute pancreatitis. Thus, the main problem in acute pancreatitis, especially in the severe form of the disease, is the difficulty of planning clinical studies capable of giving reliable statistically significant answers regarding the benefits of the various proposed therapeutic agents previously tested in experimental settings.

According to the pathophysiology of acute pancreatitis, the efficacy of the drugs already available, such as gabexate mesilate, lexipafant and somatostatin should be re-evaluated and should be probably administered in a different manner. Of course, also in this case, we need adequate studies to test this hypothesis.

In the past few years, several new therapeutic options have changed the management of acute pancreatitis; for example, therapeutic ERCP with endoscopic sphincterotomy in severe biliary pancreatitis, the use of early antibiotic treatment in necrotizing pancreatitis and the demonstration that enteral feeding is able to decrease the inflammatory response. In this paper, we describe new therapeutic treatments which could modify the current approach to acute pancreatitis in the near future. This is possible only because we have new information to better understand the pathophysiological processes of the disease.

We can distinguish three clinical phases regarding the pathophysiology of acute pancreatitis. There is not very much information on the initial phase of acute pancreatitis in humans and, for the most part, it comes from experimental studies [1]. Of course, it is obvious that we can obtain good therapeutic results only if we treat the pancreatitis as soon as possible. In the early stage of acute pancreatitis, within the first week of the start of the inflammatory process, there is an inappropriate activation of proteases which, together with microcirculatory disorders, leads to the appearance of necrosis. Subsequently, these events may be associated with macrophage activation and progress to necrosis. This is a very dangerous stage because gut and biliary bacteria can infect the necrosis during the third-fourth week of acute pancreatitis. Mortality is approximately 32% in the early stage (mainly from organ failure) and 12% in the middle stage (5% from infection). If the necrosis becomes infected, mortality is 19% in the third week and 37% in the fourth.

There is experimental and clinical evidence that the time limit for efficacious medical treatment is no more than 60 hours from the onset of the symptoms of acute pancreatitis [2]. Another important aspect in the correct approach for managing acute pancreatitis is the correct clinical classification. We should thank Bradley for his efforts in changing the classification of the disease from a pathological one to a clinical one [3]. Bradley's paper summarizes the evolution from the Marseille [4] to the Atlanta [3] classification system: the Marseille classification recognizes two morphological stages of pancreatitis: edematous acute pancreatitis and the necrotizing form and, for this reason, it is a "pathological classification". The Atlanta classification distinguishes two forms from a clinical point of view, including mild acute pancreatitis, without any complications, and severe pancreatitis characterized by systemic and local complications such as necrosis, pseudocysts and distant organ failures.

As in other diseases, the pathophysiological aspects of acute pancreatitis should guide our therapeutic approach. On the other hand, we should also consider that the treatment needs to be tailored to each individual patient and we should also take into account the available resources of each Institution.

Since 1994, many papers have been published suggesting good medical therapy for patients with acute pancreatitis [3,5-12], but unfortunately there is no congruence in the various guidelines regarding stratification of severity, diagnosis, treatment and presence of Pancreas Units [13]. In the same way, there are no homogeneous evidence levels in the various guidelines [13]. These differences are quite surprising because most of the participants are the same experts who decide on the various guidelines. In addition, as suggested by Bradley [13], there is the need to unify the various guidelines. In brief, there is the need to address the efforts of the guideline writers in order to unify the various guidelines. One example of the rapid evolution of the knowledge of acute pancreatitis is the following: the UK guidelines were released in 1998 [5], revised in 2005 [6] and, after just a few weeks, some researchers asked to change the new 2005 UK guidelines [14]. Another problem with the guidelines is that many clinical practitioners in the same country follow different guidelines [15] and others do not fully apply them in clinical practice [16]; moreover, in most of the guidelines, the basic management of acute pancreatitis is not reported: some examples are control of pain and control of nausea, vomiting and ileus. First of all, there are no extensive studies on the pharmacological control of pain in acute pancreatitis [17-20]; this is quite surprising due to the importance of this symptom. Second, there are many therapeutic procedures performed on patients who are not included in the practical guidelines: for example, continued gastric suction is often used in treating patients with acute pancreatitis, even if most of the published studies limit this approach only to patients with severe disease [21-23]. Finally, gastric acid secretion inhibition is largely used in patients with acute pancreatitis, even if there are very few studies on this issue and the results are not conclusive [24,25]. The reason for these discrepancies is that there is poor homogeneity in the treatment of acute pancreatitis.

Regarding the experimental perspectives in the treatment of acute pancreatitis, we would point out that, in the last five years, more than 2000 papers on the treatment of acute pancreatitis in experimental models have been published. About a half of these were carried out on edematous pancreatitis and only a few of the substances tested have been applied in clinical practice. One of these substances is infliximab, a monoclonal TNF-antibody. It was tested in 100 rats randomly assigned to 10 groups [26]. In acute edematous pancreatitis and in severe necrotizing pancreatitis, the drug significantly decreased serum amylase activity and the histopathological score; moreover, in the severe forms with necrosis, infliximab ameliorated both parenchymal and fatty tissue necrosis of the pancreas and it also alleviated alveolar edema and ARDS-like pulmonary complications, even if this difference was not significant. Another avenue investigated was that of antioxidant treatment to avoid necrosis as a result of the fall in cytokine levels. Thus, a particular molecule has been studied for its antioxidative properties: resveratrol [27]. It was evaluated in acute pancreatitis induced by tert-butyl hydroperxide injection. Changes in the pancreata were much less pronounced in the rats which received resveratrol for 8 days prior to injection. In this way, it seems that pancreatic cells may be prevented from undergoing structural changes during the experimentally-induced acute inflammation; we would point out that antioxidant treatment for acute pancreatitis is a never ending story. However, the utility of such experimental models may have some limitations and a full extrapolation of experimental data from laboratory animals to humans must be done with caution; a paper published in 2001 highlighted the limitations of experimental models in acute pancreatitis [28]. In this regard, we report the example of interleukin-10. In experimental studies, this molecule was effective [29] in reducing the severity of acute pancreatitis, but it was not capable of preventing new organ failures in a clinical setting [30]. On the other hand, polyunsaturated fatty acids were able to decrease the severity of experimental acute pancreatitis and these substances were able to reduce the length of hospitalization and the duration of jejunal feeding in humans, even if they were not able to decrease the number of new complications [31,32]. What are the problems in carrying out studies on therapeutic agents in acute pancreatitis? In the last 5 years, only 11 studies have been published on the treatment of severe human acute pancreatitis and most of them regard early antibiotic treatment. This happens because it is difficult to plan clinical studies on acute pancreatitis capable of giving specific answers regarding the benefits of the various therapeutic agents proposed in the human clinically severe form of the disease. Furthermore, there is no translational research in this field. There is the need to better design future clinical trials in acute pancreatitis [33]. In fact, therapeutic trials need to record the time from the onset of symptoms to intervention and there is the need of using widely accepted prognostic indices to categorize the severity of acute pancreatitis. The end-points to use must be relevant and interpretable; mortality is important but more work is necessary in developing patient outcomes. Good alternatives include the measurement of permanent target organ damage, disability, quality of life, pain scores, category of intervention, surgery, hospital stay and return to work, and including patients with a single etiology of acute pancreatitis or at least only those with a predominant etiology of the disease in the specific country. The role of immune-modulation is shown by lexipafant in a study including 290 patients: 151 were in the lexipafant group and 139 in the placebo group. Four patients were subsequently excluded (three due to an incorrect diagnosis and one due to a major violation of the protocol). The analysis of complications regarded 138 patients in the placebo group and 148 in the lexipafant group. The analysis of attributable mortality was carried out in 147 patients of the lexipafant group and in 136 in the placebo group. The analysis of treatment performed within 48 hours from the onset of the symptoms of acute pancreatitis was performed in 104 patients of the lexipafant group and in 95 patients of the placebo group. This study, performed with an adequately sized sample, has shown that the antagonism of PAF activity on its own is not sufficient to ameliorate the systemic inflammatory response syndrome in severe acute pancreatitis: however, if we look at the data reported, we cannot exclude the possibility that lexipafant may have some effect, especially in patients treated within 48 hours from the onset of symptoms which regard a reduction in the appearance of pseudocysts and deaths [34].

The trials with infliximab are an example of the "magic bullet" approach which has typified anti-cytokine trials. The restoration of homeostasis with a single intervention belies the complex and coordinated nature of the inflammatory response. Deleterious effects have been recorded when single proximal mediators of the inflammatory response were blocked: development of anti-DNA antibodies, antinuclear antibodies, anticardiolipin antibodies, antithyroid antibodies, appearance of systemic lupus erythematosus, neurological signs and symptoms associated with demyelinating lesions of the central nervous system, thyroid dysfunction, arthritis, myositis, systemic sclerosis, pemphigus vulgaris, vitiligo and carpal tunnel syndrome. In clinical practice, there is the necessity of not using "magic" drugs alone; we need more drugs capable of involving the different aspects of the disease [35]. Furthermore, we must be aware of several autoimmune phenomena in patients treated with cytokine and anticytokine therapy [36]; we also need to change the way the results of drug trials are communicated to the medical world [35]. Our needs are mainly the following: a correct scientific method (clinical trials should be preceded by experimental and pilot studies in order to confirm the safety and the correct dosage and to estimate the necessary efficacy of future trials); improvement of the communication of the results; the editors must share the responsibility of publishing well-designed and well-conducted clinical studies whether or not the results are negative and commercial influence (the risks associated with dealing with biotechnology companies are well-known). Companies can be under severe pressure to repay the venture capitalists and shareholders. Thus, there is the need for independent monitoring of the data and safety in company-sponsored clinical trials. One example of this assumption may be the highly debated efficacy of protease inhibitors in human acute pancreatitis [37]. Ten articles of randomized controlled trials evaluating the effects of protease inhibitors (aprotinin and gabexate) for acute pancreatitis were retrieved by systematically searching Medline and the Cochrane Library and Ovid databases published between January 1966 and December 2003. The main outcome of interest was the overall mortality rate from acute pancreatitis. When protease inhibitors were given to patients with mild pancreatitis, the results were not significant; on the other hand, when these proteins were given to patients with severe pancreatitis, the mortality rate decreased significantly. Several steps may be blocked at the same time and this might be achieved by using several drug combinations at the same time or by the multiple action of a single drug in order to block the protease cascade as well as the cytokine cascade [2].

Another important aspect for the treatment of acute pancreatitis is the prevention of the infection of the pancreatic necrosis. Enteral feeding plays an important role in this. A study of 34 severe acute pancreatitis patients shows that systemic inflammatory response syndrome, sepsis, organ failure, and ICU stay were globally improved in the enterally-fed patients. The acute phase response and disease severity scores (C-reactive protein, APACHE II) were significantly improved following enteral nutrition without any change in the computed tomography scan scores. Thus, the conclusion was that enteral feeding modulates the inflammatory and sepsis response in acute pancreatitis and is clinically beneficial. This is the first clinical study demonstrating the beneficial effect of enteral nutrition in decreasing the inflammatory and sepsis response in severe pancreatitis [38]. There is no doubt that it is better to administer enteral feeding via a gastric tube than via a jejunal tube [39]. In an another study, a total of 50consecutive patients with objectively graded severe acute pancreatitis were randomized to receive either gastric or jejunal feeding via a fine bore feeding tube. A total of 27 patients were randomized to gastric feeding and 23 to jejunal feeding. Clinical differences between the two groups were not significant. Overall mortality was 24.5% with five deaths in the gastric group (18.5%) and seven in the jejunal group (31.8%). The simpler, cheaper, and more easily used gastric feeding is as good as jejunal feeding in patients with objectively graded severe acute pancreatitis. This appears to be a useful and practical therapeutic approach to enteral feeding in the early management of patients with severe acute pancreatitis. There is also no doubt that probiotics associated with enteral feeding may become an alternative therapy replacing early antibiotic use to prevent infection in severe pancreatitis [40]. In this regard, there is a study planned as a double-blind placebo-controlled randomised multicenter trial in which patients will be randomly allocated to a multispecies probiotic preparation (Ecologic 641) or a placebo; it will be carried out in 15 Dutch Hospitals. The substance being studied is administered twice daily through a nasojejunal tube for 28 days or until discharge. The inclusion criteria are the following: adult patients with a first onset of predicted severe acute pancreatitis (Imrie criteria 3 or more, CRP 150 mg/L or more, APACHE II score 8 or more) and the exclusion criteria are post-ERCP pancreatitis, malignancy, infection/sepsis caused by a second disease, intra-operative diagnosis of pancreatitis and use of probiotics during the study. The substance being studied administration starts within 72 hours after onset of the abdominal pain. The primary endpoint is the total number of infectious complications; the ancillary endpoints are mortality, necrosectomy, antibiotic resistance, hospital stay and adverse effects of the probiotics. A sample size of 200 patients was calculated to demonstrate that probiotic prophylaxis reduces the proportion of patients with infectious complications from 50% to 30%, with alpha 0.05 and power 80%. We are awaiting the results of this study in order to draw a final conclusion on the effectiveness of probiotic prophylaxis in preventing septic complications in severe acute pancreatitis [41].

Regarding antibiotic therapy, a meta-analysis performed by Sharma et al. has recently been published which shows the need for using early antibiotic therapy in order to prevent sepsis and mortality in severe acute pancreatitis [42]. They have shown that antibiotic prophylaxis decreases sepsis and mortality in patients with acute necrotizing pancreatitis and they suggested that all patients with acute necrotizing pancreatitis should receive prophylaxis with an antibiotic of proven efficacy. The authors concluded that all patients with acute necrotizing pancreatitis should receive early antibiotic treatment [42]. It is clear that not all researchers agree that severe acute pancreatitis should be treated with early antibiotic administration [43]. After the publication of the paper of Isenmann R. et al. [43], a discussion of its validity was begun [44,45]. The main criticisms were that: the pancreatic necrosis was confirmed by CT criteria in only 58 patients, 5 patients had Staphylococcus epidermidis coagulase negative strains and the dectection of this species might be considered more a contamination than a true infection. Once the presence of infected necrosis was determined, it was not clear if surgical intervention was immediate or if this was preceded by the open administration of antibiotics; 28% of the antibiotic-treated patients and 46% of the patients in the placebo group had received an open treatment with antibiotics. These data could suggest not only the need, but the inevitability, in everyday clinical practice, of prescribing early antibiotic treatment in the management of severe necrotizing pancreatitis, either prophylactically or "on demand". Moreover, why did the authors choose an antibiotic such as fluoroquinolones which, in a previous clinical study, did not demonstrate efficacy similar to imipenem? And how many patients were fed enterally? [45]. These questions remain unanswered in their paper.

Another open question in the treatment of acute pancreatitis is refeeding. It is crucial in patients who have recovered from an acute episode of pancreatitis, but there are very few studies on this issue. From a practical point of view, Levy et al. [46] have proposed the following formula in predicting the pain during refeeding: 0.64 a + 1.11 b + 2.18 c – 9.06, where a = Balthazar's CT score, b = duration of painful period, c = serum lipase concentration on the day before refeeding <3 times the upper normal limit and 9.06 = constant. To prevent an acute relapse of acute pancreatitis after oral refeeding, the use of lanreotide has been suggested [47]. In a French study, only 4.3% of the patients treated with Lanreotide had a recurrence of pain from acute pancreatitis, but 65.2% experienced adverse effects from the drug [47]. Since this is an uncontrolled pilot study, the results should be taken with caution and should be further confirmed through a double-blind controlled study. From a practical point of view, we also need to know the exocrine pancreatic function after an acute episode of pancreatitis in order to cure possible maldigestion. There are very few studies exploring this aspect [48-51]. In the our study [51], patients with acute pancreatitis were studied using the secretin-cerulein test after acute alcoholic or biliary pancreatitis; pancreatic insufficiency was significantly more frequent and more severe in alcoholic pancreatitis than after acute biliary pancreatitis. These findings, together with the fact that the insufficiency was also more persistent, suggest that acute alcoholic pancreatitis may occur in a pancreas which already has chronic lesions. Thus, in patients with alcoholic pancreatitis, there is the need for enzyme supplementation during refeeding and this aspect represents an important issue in nutritional support; however, there are no specific studies showing the efficacy of enzyme oral supplementation.

In conclusion, the cornerstones for the correct treatment of acute pancreatitis are those reported in the published guidelines, but we need hard work to further develop our knowledge in this fascinating field.
